# The Association between Survival and the Pathologic Features of Periampullary Tumors Varies over Time

**DOI:** 10.1155/2014/890530

**Published:** 2014-07-01

**Authors:** Jennifer K. Plichta, Anjali S. Godambe, Zachary Fridirici, Sherri Yong, James M. Sinacore, Gerard J. Abood, Gerard V. Aranha

**Affiliations:** ^1^Department of Surgery, Loyola University Health Systems, 2160 S. First Avenue, Maywood, IL 60153, USA; ^2^Department of Pathology, Loyola University Health Systems, 2160 S. First Avenue, Maywood, IL 60153, USA; ^3^Stritch School of Medicine, Loyola University Medical Center, 2160 S. First Avenue, Maywood, IL 60153, USA; ^4^Department of Preventive Medicine, Loyola University Medical Center, 2160 S. First Avenue, Maywood, IL 60153, USA

## Abstract

*Introduction*. Several histopathologic features of periampullary tumors have been shown to be correlated with prognosis. We evaluated their association with mortality at multiple time points. *Methods*. A retrospective chart review identified 207 patients with periampullary adenocarcinomas who underwent pancreaticoduodenectomy between January 1, 2001 and December 31, 2009. Clinicopathologic features were assessed, and the data were analyzed using univariate and multivariate methods. *Results.* In univariate analysis, perineural invasion had a strong association with 1-year mortality (OR 3.03, CI 1.42–6.47), and one lymph node (LN) increase in the LN ratio (LNR) equated with a 5-fold increase in mortality. In contrast, LN status (OR 6.42, CI 3.32–12.41) and perineural invasion (OR 5.44, CI 2.81–10.52) had the strongest associations with mortality at 3 years. Using Cox proportional hazards, perineural invasion (HR 2.61, CI 1.77–3.85) and LN status (HR 2.69, CI 1.84–3.95) had robust associations with overall mortality. Recursive partitioning analysis identified LNR as the most important risk factor for mortality at 1 and 3 years. *Conclusions.* Overall mortality was closely related to the LNR within the first year, while longer follow-up periods demonstrated a stronger association with perineural invasion and overall LN status. Therefore, the current staging for periampullary tumors may need to be updated to include the LNR.

## 1. Introduction 

Several histopathologic features of periampullary adenocarcinoma tumors correlate with survival following resection, including lymph node (LN) status, perineural infiltration, lymphovascular invasion, and lymph node ratio (LNR). Both perineural infiltration and lymphovascular invasion in pancreaticoduodenectomy specimens were found to be associated with a decreased 5-year survival [1]. Perineural invasion alone has also been shown to be a strong predictor of survival in patients with periampullary, duodenal, and ampullary adenocarcinomas [2–4]. Talamini et al. identified a higher resectability rate and better prognosis in patients with ampulla of Vater tumors and emphasized that the LN status likely influenced survival outcomes [5]. More recently, the utility of the LNR, defined here as the number of positive LN divided by the total number of LN assessed in a surgical specimen, has been highlighted as a potential factor in predicting mortality [6, 7].

For nonperiampullary tumors, the LNR has also been correlated with prognosis, including gastric cancer [8], esophageal squamous cell carcinoma [9], small bowel adenocarcinoma [10], colorectal cancer [11], breast cancer [12], and melanoma [13]. Notably, the LNR was an independent prognostic indicator for overall survival in patients undergoing curative gastrectomy for gastric cancer, but it did not prove to be superior to standard pN staging [14]. In contrast, the LNR in patients with node-positive breast cancer was able to further subdivide patients across all pN groups, suggesting that the LNR may add prognostic value to the traditional TNM classification [15]. Furthermore, the LNR may be a more precise predictor of survival than traditional pN staging in some patients with colon cancer [16, 17]. In patients with cholangiocarcinoma, LN metastasis serves as a major prognostic factor, while the number of LN resected and the LNR also yield high prognostic value [18, 19]. Considering this, the LNR has been proposed as a superior prognostic variable for numerous types of tumors.

As such, the association between the LNR and periampullary tumors has also been investigated. Following curative resection for ampulla of Vater carcinoma, the LNR and a minimum of 16 evaluated nodes were identified as robust prognostic factors for disease-specific survival [20]. In contrast, retrospective evaluations of pancreatic cancer and ampullary carcinoma demonstrated that the number of metastatic nodes, but not LNR, was one of the most important prognostic factors [21, 22]. However, a significant association between the LNR and survival for patients with pancreatic cancer was identified in separate studies [6, 23–25]. Furthermore, using data from patients undergoing pancreaticoduodenectomy for pancreatic adenocarcinoma, the LNR has been shown to be one of the most powerful predictors of short- and long-term survival [25] and has been suggested as a new tool for stratifying patients in future trials [6]. Thus, beyond the qualitative LN status (positive or negative nodes), the LNR may provide a quantitative tool that improves the current classification system for periampullary tumors [7, 26].

Although most of the aforementioned studies evaluated the association between various histopathologic features and prognosis, they were unable to instigate significant changes in the staging classification for periampullary tumors. This outcome was likely attributable to the fact that their focus was often seeking only one variable as the best predictor of their outcome, as opposed to utilizing several criteria similar to the current TNM staging to better classify periampullary tumors. Therefore, we aim to evaluate the association between mortality and several histopathologic features of periampullary adenocarcinoma tumors, including the LNR, at multiple time points in order to better predict patient prognosis.

## 2. Methods

We performed a retrospective review to assess the correlation between several histopathologic features of periampullary adenocarcinoma tumors and mortality following surgical intervention. We identified 207 patients with periampullary adenocarcinoma tumors who underwent attempted curative resection (pancreaticoduodenectomy, R0 or R1 resection completed) between January 1, 2001 and December 31, 2009. Patients with concurrent malignancies, a history of periampullary adenocarcinoma (or other pancreatic cancers), or perioperative mortalities (i.e., patients dying within 30 days of surgery) were excluded. The Social Security Death Index was utilized to determine current living status (last updated at April 27, 2012). Clinical and histopathologic features were assessed from the medical record, and overall survival at 1 year, 3 years, and to date was determined. Although pathology and operative reports were available for all patients, more detailed records were not routinely uploaded into our electronic medical record until 2006, which limited the collection and utilization of some clinical parameters. The variables considered in our study were the most consistently reported. Disease-free survival was unable to be calculated due to the limited follow-up at our institution. This study was approved by the Loyola University Health Systems Institutional Review Board.

### 2.1. Statistical Analysis

Statistical analyses were conducted using Stata 10.0 (StataCorp, College Station, TX). Categorical variables were analyzed using Chi-squared (*χ* ^2^) tests, and continuous variables were analyzed using Mann-Whitney *U* tests. Statistical significance was defined as *P* ≤ 0.05  (2-sided). Univariate and multivariable logistic regression were performed to assess clinicopathologic characteristics associated with 1- and 3-year mortality following surgical resection (odds ratios and 95% confidence intervals reported). The selection of variables for the multivariate analyses was based upon the results of the univariate analyses. Similarly, univariate and multivariate Cox proportional hazard model analyses were performed to evaluate the relationship of these features with all mortality to date (hazard ratios and 95% confidence intervals reported). The variables were selected based upon the results of the logistic regression analyses.

Classification and Regression Trees (CART 6.0; Salford Systems, San Diego, CA) were used to analyze the interactions between 11 different risk factors and the outcomes of interest and 1- and 3-year mortality. The risk factors included age, gender, subtype of periampullary tumor, tumor size, pathologic margin status, LN status, total number of LN removed, number of positive LN, LNR (number of positive LN/total number of LN removed), perineural infiltration, and lymphovascular invasion. CART analysis was used to grow a decision tree using the Gini splitting criteria with a minimum number of 10 parent node cases and a minimum number of cases for the child nodes of 1. Given the limited size of the data set (*n* = 207), the tree's classification accuracy was determined by way of a cross-validation method. To do this, the data were allotted (i.e., jackknifed) into five segments. One segment was successfully held out while the remaining segments were used to grow a tree, and the classification accuracy of the holdout segment was recorded. The overall cross-validation accuracy was determined by summing the results across all of the jackknifed segments.

## 3. Results

Of the 207 patients identified, there were 106 males and 101 females with a median age of 69 years (range 28–87 years). There were 17–28 surgeries performed annually (median 23 surgeries) between 2001 and 2009. Most tumors were pancreatic in origin (56% versus 23% ampullary, 12% duodenum, and 9% distal common bile duct). Similar proportions were noted in a cohort of patients from the SEER cancer registry who underwent pancreaticoduodenectomy between 1993 and 2003: 62.5% pancreatic, 18.9% ampullary, 7% duodenal, and 11.6% distal bile duct [27]. The median tumor size was 2.75 cm, and an R0 resection was achieved in 70.5% of patients (*n* = 146). Lymphovascular invasion was noted in 51% of cases (*n* = 106), and perineural infiltration was reported in 66% (*n* = 137). The median number of LN identified in the surgical specimen was 19 LN. At least one LN was positive in 64% of patients (*n* = 133), and the median number of positive LN was one. The median LNR was 7.7%. While 207 patients were followed up for at least 1 year, only 187 had been followed up for at least 3 years at the time of analysis. At 1-year follow-up, significant differences between survivors and nonsurvivors were noted for 8 clinicopathologic features (age, tumor size, margin status, lymphovascular invasion, perineural invasion, overall LN status, number of positive LN, and LNR; Table 1). Excluding age, similar differences were observed between the two groups at 3-year follow-up (Table 1). The median overall follow-up was 1.9 years, while it was 5.6 years for survivors alone and 1.7 years for nonsurvivors alone. The crude overall survival was 31% at the end of the follow-up period. Overall survival at 1 year was 73% and dropped to 40% by 3 years after surgery.

Using univariate logistic regression, 1-year mortality was independently associated with 7 clinicopathologic characteristics: age, tumor size, margin status, lymphovascular invasion, perineural infiltration, LN status, and LNR (data not shown). More specifically, perineural invasion had the strongest association with 1-year mortality (OR 3.03, CI 1.42–6.47), although LN status (OR 2.93, CI 1.41–6.1) and margin status (OR 2.87, CI 1.5–5.49) were quite similar. Additionally, an increase in the LNR by 1% increased the odds of mortality by 1.03-fold. However, the average number of LN removed was 20; thus, a change by 1 LN would equate with a 5% change in the LNR and thus a 1.16-fold increase in the odds of mortality. Multivariate analysis also revealed a significant association between 1-year mortality and the LNR (OR 1.02, CI 1–1.04; Table 2). Notably, the model adjusting for the LNR (model E) accounts for 13.3% of the variability (adjusted *R* ^2^) in 1-year mortality, while the other models account for 10.8–12.9% (Table 2).

For 3-year mortality, univariate logistic regression analyses revealed independent associations with 6 clinicopathologic characteristics: tumor size, margin status, lymphovascular invasion, perineural infiltration, LN status, and LNR (data not shown). In contrast to 1-year mortality where perineural invasion was strongest, at 3 years the overall LN status (positive or negative) had the strongest association (OR 6.42, CI 3.32–12.41). However, perineural invasion remained a strong predictor (OR 5.44, CI 2.81–10.52). Similar to 1-year mortality, an increase in the LNR by 1% increased the odds of mortality by 1.08-fold. Thus, a change by 1 LN would equate with a 5% change in the LNR (assuming 20 LN were assessed) and consequently a 1.47-fold increase in the odds of mortality. Therefore, qualitative LN status and perineural invasion appear to be stronger predictors than LNR in predicting 3-year mortality. This is further supported by multivariate analyses where the model adjusting for overall LN status (model D) accounts for 22.5% of the variability in 3-year mortality (adjusted *R* ^2^), while the other models account for 12.9–20.6% (Table 2). Similar findings were noted using univariate Cox proportional hazards, where perineural infiltration (HR 2.61, CI 1.77–3.85) and overall LN status (HR 2.69, CI 1.84–3.95) had strong associations with overall mortality (data not shown). In multivariate Cox analyses, all clinicopathologic characteristics included were significant independent predictors (*P* < 0.05) in all models (Table 3). More specifically, the presence of positive LN appeared to have the strongest crude association with overall mortality.

### 3.1. CART Analysis for 1- and 3-Year Mortality

To create the CART decision trees, 11 risk factors were entered into the software to classify survivor and nonsurvivor patients at 1- and 3-year follow-up. Variables included age (continuous), gender (male or female), tumor size (continuous), margin status (positive or negative), tumor subtype (pancreatic, distal common bile duct, ampullary, or duodenal), lymphovascular invasion (positive or negative), perineural invasion (positive or negative), LN status (positive or negative), number of positive LN (continuous), total number of LN removed (continuous), and LNR (continuous).

**Figure 1:**
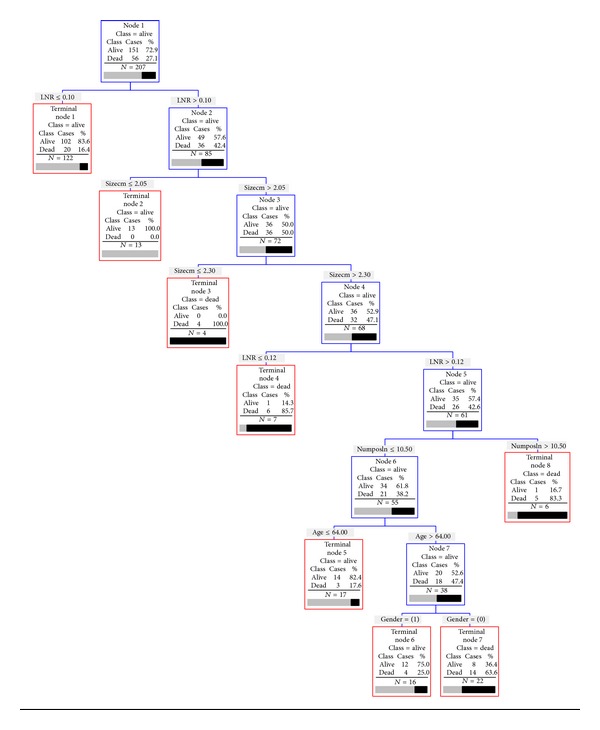
Results of recursive partitioning analysis to predict 1-year mortality. Numposln: number of positive lymph nodes; Sizecm: tumor size in centimeters; LNR: lymph node ratio.

For 1-year mortality, the CART tree grown with the training data set contained 7 levels (Figure 1). The most important factor was the LNR as 84% with a LNR ≤ 0.1 were alive at 1 year (*n* = 102 of 122). Of patients with a LNR > 0.1, the next most important risk factor was tumor size, where 100% of patients with tumors ≤2.05 were alive at 1 year (*n* = 13) and 100% of those with tumors >2.05 but ≤2.3 died (*n* = 4). Further splits were developed, and the decision tree had an overall classification accuracy of 82% for the training data set. To validate these findings, a subset of the data was used to test the model, yielding a score of 74% overall accuracy (Table 4).

**Figure 2:**
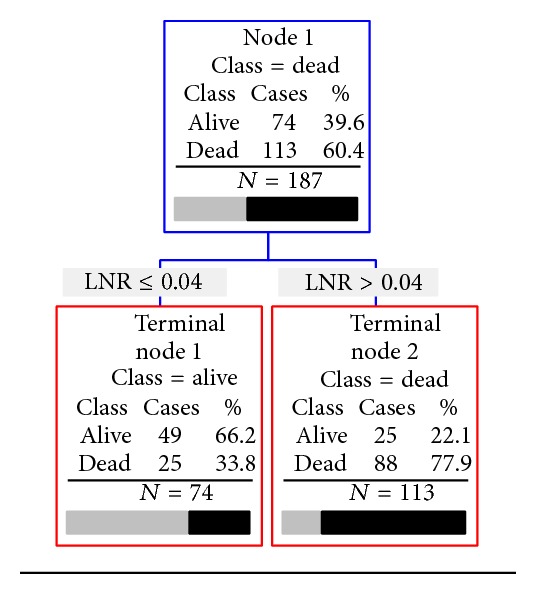
Results of recursive partitioning analysis to predict 3-year mortality.

For 3-year mortality, the CART tree grown with the training data set contained only one level (Figure 2). The most important factor was again the LNR as 78% with a LNR > 0.04 were deceased at 3 years (*n* = 88 of 113) and 66% of those with a LNR ≤ 0.04 were alive (*n* = 49 of 74). This decision tree had an overall classification accuracy of 73% for the training data set, which was similar for the testing data set (overall accuracy 72%; Table 4).

## 4. Discussion

Based on a similar cohort of patients undergoing pancreaticoduodenectomies from 1998 to 2007 at our institution, we previously demonstrated an inverse relationship between the LNR and survival, which was strongest for pancreatic and ampullary tumors [7]. Here, we again demonstrate that a higher LNR is likely a significant risk factor for patients undergoing attempted curative resection of a periampullary adenocarcinoma tumor. Using multiple analytic methods, it proved to be a significant variable in univariate and multivariate regression analyses, as well as being identified as the best initial stratification variable in recursive partitioning analysis. More specifically, the CART analyses suggest that the two most important risk factors for determining 1-year mortality were the LNR and tumor size, while only the LNR was able to risk-stratify patients at 3 years. A focused, separate analysis of 246 patients with specifically pancreatic adenocarcinoma reported a significant prognostic value of the LNR for both short- and long-term survival after PD [25]. This was similarly confirmed in a recent study of 551 patients who underwent resection for periampullary tumors, and a LNR > 0.2 was identified as an independent prognostic factor for overall survival [28]. While our analyses included the LNR as a continuous variable, one of the original studies evaluating the association between the LNR and pancreatic cancer found a statistically significant difference only for patients with a ratio of 15% to 19% [29], which has subsequently been used as the categorical cutoff for other follow-up studies [7, 28]. The slight difference in cutoff values between the earlier report and the current investigation may be related to the inclusion of all periampullary tumors here.

In this study, we wished to determine whether LNR by itself or other risk factors influenced survival in all four periampullary adenocarcinoma tumors over a longer period of time. We demonstrated that several other histopathologic features appeared to be significantly associated with prognosis, including tumor size, margin status, qualitative LN status, perineural infiltration, and lymphovascular invasion. Data on tumor grade and adjuvant therapies were incomplete and, thus, not included in our analyses. Excluding the LNR, perineural invasion appeared to be most significantly associated with 1-year mortality, while overall LN status yielded a stronger correlation at 3-year follow-up, although both variables were significant at both time points. In a similar study of 346 patients undergoing resection for periampullary cancers, only nodal metastasis and neural invasion significantly predicated overall survival in multivariate analysis [30]. Nevertheless, as noted in a recent study of 1,147 patients over three decades, long-term survival has not significantly improved for patients undergoing resection for pancreatic cancer [31], which highlights the importance of creating novel stratification systems to help develop targeted and more appropriate treatment regimens.

It has been proposed that the subtype of periampullary tumor also contributes to prognosis [32, 33]. Therefore, identification of biomarkers that aid in distinguishing the various subtypes may consequently correlate with survival. For example, recent investigations have demonstrated that hepatocyte nuclear factor 4-alpha (HNF4*α*) is an effective tool for identifying different ampullary cancer subtypes and is an independent predictor of a favorable prognosis [34]. Although limited by a relatively small sample size, subdividing our patient population by tumor subtype did not appear to influence patient stratification in our recursive partitioning analysis.

Others have suggested that a minimum number of LN need to be assessed in the surgical specimen in order to optimize prognostic accuracy and prevent stage migration errors. Using a cohort of 5,465 patients from the SEER cancer registry that underwent pancreaticoduodenectomy between 1993 and 2003, Gutierrez et al. demonstrated that a minimum of 10 LN should be examined in order to determine LN status [27]. Here, the mean number of LN assessed was 19.8, but there was one patient with only 1 LN identified in the surgical specimen (per report) and 17 patients with fewer than 10 LN reported. Of this patient subset, 1-year survival was 53% versus 74.7% for patients with ≥10 LN assessed (*P* = 0.053). By 3-year follow-up, this initially notable difference disappeared (3-year survival 41.2% for those with <10 LN versus 39.4% for those with ≥10 LN). Furthermore, there was no significant difference overall between 1- or 3-year survivors and nonsurvivors based on the total number of LN assessed (Table 1). These findings suggest that the total number of LN evaluated may be an early risk factor but likely becomes less important as time progresses.

One of this study's strengths and weaknesses is the inclusion of patients over a 9-year period. During this time, the analysis of the pathologic specimens likely evolved with the emerging data related to margin status. For example, a study by Verbeke et al. was one of the first to evaluate the implementation of a standardized protocol for assessing resection margins in pancreatic head adenocarcinomas and found a significantly higher R1 rate with the newer protocol (R1 rate 85% with the standardized protocol versus 53% with the nonstandardized protocol) [35]. The R1 rates, however, were not significantly altered by the implementation of the standardized protocol for ampullary or distal bile duct cancers [35]. The R1 rates for all periampullary adenocarcinoma tumors in this study varied from 7% in 2008 to 56.5% in 2005, while the most recent rate in 2009 was 23% and the overall was 29%. Furthermore, the resection margin status was significantly associated with mortality in our regression analyses. Although it is clear that specimen dissection technique and standardization of the pathologic examination are crucial [36, 37], the advancements in imaging over recent years have also influenced the selection of patients appropriate for attempted surgical resection [38] and, thus, the overall margin status and ability to achieve an R0 resection.

In addition to the more traditional histopathologic features assessed in surgical specimens, current research is investigating other potential biomarkers that may better correlate with prognosis and/or potentially aid in early diagnosis. Cancer antigens CA19-9 and CA125 were some of the earlier biomarkers to be evaluated, but they lack sensitivity and specificity to be used for predicting prognosis [39]. Currently, serum CA19-9 levels are used primarily for diagnosis and/or following patients with active or a history of pancreatic cancer [39, 40]. In an attempt to find novel biomarkers of the disease, one study found that CD56 and certain mucins were associated with vascular and perineural invasion and together may serve as markers of prognosis in patients with periampullary tumors [41]. Cyclin D1 was also found to be independently associated with prognosis in some periampullary tumors [42, 43], while p16 protein has shown some correlation with perineural invasion and, thus, potentially prognosis [44]. Although some of these biomarkers show great promise, their exact utility in prognosticating outcomes has yet to be validated in routine clinical practice.

In contrast, the information needed to calculate the LNR of a surgical specimen is often readily available in most pathology reports. Furthermore, it has the potential to serve as an adjunct to traditional TNM staging and may have additive risk stratification capability, which may be particularly important early in the disease course when survival declines most rapidly. Our study systematically evaluated this and several other histopathologic features at multiple time points in order to adequately assess the ability of these variables to risk-stratify these tumors. In addition, our findings are supported by a unique and thorough analysis, which not only included traditional multivariate regression analyses, but also were further verified by another underutilized technique called recursive partitioning. The benefits of the latter method are its capacity to consider numerous variables simultaneously (even more than that typically recommended for multivariate analyses), its ability to consider one variable in the context of other variables, and the mathematical calculations performed to determine the best stratification variable for the specified outcome within a particular data set. While regression methods may be more useful when seeking to quantify the relative contribution of the explanatory variables, recursive partitioning often provides insight into the data structure and relationships between variables. This analytical method has been used previously for similar questions [45, 46], but our study is one of the first to apply this technique as a tool for risk-stratifying patients with periampullary tumors. As suggested by Cook and Goldman, this simple and intuitive type of analysis for classifying subjects has the potential to help identify novel and synergistic interactions among multiple variables and potentially aids in developing more practical risk stratification tools [47].

## 5. Conclusions

Overall mortality appears to be more closely related to the LNR within the first year following surgery. Longer follow-up periods, however, demonstrated a stronger association between overall mortality and the qualitative LN status and perineural invasion. Evidence suggests that the current staging paradigm for periampullary adenocarcinoma tumors may need to be updated to include the LNR. However, further investigations are required to fully evaluate the utility of the LNR as either a replacement or an adjunct to the standard pN staging. In addition, patients living beyond a certain time frame following curative resection may require reanalysis for determining their continued prognosis.

## Figures and Tables

**Table 1:** Clinicopathologic characteristics stratified by survival at one and three years.

Covariate	1 year				3 years			
	Overall	Alive	Dead	*P*	Overall	Alive	Dead	*P*
Age (years)								
Median	69	68	71	**0.014**	69	67.5	70	0.064
Range	28–87	28–87	30–87		28–87	28–87	30–87	
Gender								
Male	106	82	24	0.143	97	42	55	0.279
Female	101	69	32		90	32	58	
Tumor size (cm)								
Median	2.75	2.5	3.2	**0.001**	2.75	2.3	3	**<0.001**
Range	0.4–8.5	0.4–6.5	1–8.5		0.4–8.5	0.4–5.2	0.8–8.5	
Margins								
Negative	146	116	30	**0.001**	130	64	66	**<0.001**
Positive	61	35	26		57	10	47	
Lymphovascular invasion								
Negative	101	81	20	**0.022**	93	53	40	**<0.001**
Positive	106	70	36		94	21	73	
Perineural invasion								
Negative	70	60	10	**0.003**	62	41	21	**<0.001**
Positive	137	91	46		125	33	92	
Overall LN status								
Negative	74	63	11	**0.003**	67	45	22	**<0.001**
Positive	133	88	45		120	29	91	
Positive LN								
Median	1	1	3	**<0.001**	1	0	2	**<0.001**
Range	0–21	0–18	0–21		0–21	0–11	0–21	
Total LN assessed								
Median	19	19	19	0.598	19	18.5	19	0.656
Range	1–45	1–45	4–41		1–45	4–45	1–41	
LNR								
Median	0.077	0.056	0.141	**<0.001**	0.077	0	0.118	**<0.001**
Range	0-1	0-1	0–0.75		0-1	0–0.733	0-1	

LN: lymph nodes; LNR (lymph node ratio) = (number of positive LN)/(total LN removed) ∗ 100.

**Table 2:** Multivariate logistic regression analyses between clinicopathologic features and one- and three-year mortality following surgical resection.

Covariate	1-year mortality				3-year mortality			
	Odds ratio	95% CI	*P*	*Pseudo R* ^2^	Odds ratio	95% CI	*P*	*Pseudo R* ^2^
Model A								
Age	1.04	1–1.08	0.027	0.108	1.02	0.99–1.05	0.225	0.129
Tumor size	1.51	1.17–1.95	0.001		1.56	1.19–2.04	0.001	
Margin status	2.64	1.34–5.2	0.005		4.14	1.89–9.08	<0.001	
Model B								
Age	1.04	1.01–1.08	0.022	0.123	1.02	0.99–1.06	0.144	0.206
Tumor size	1.53	1.18–1.98	0.001		1.6	1.21–2.12	0.001	
Margin status	2.32	1.16–4.65	0.017		3.3	1.44–7.55	0.005	
Lymphovascular invasion	1.91	0.96–3.82	0.066		4.51	2.24–9.07	<0.001	
Model C								
Age	1.04	1–1.08	0.026	0.122	1.02	0.99–1.05	0.168	0.195
Tumor size	1.49	1.15–1.93	0.003		1.62	1.21–2.16	0.001	
Margin status	2.06	1–4.25	0.052		2.5	1.08–5.81	0.032	
Perineural invasion	2.09	0.91–4.84	0.083		4.37	2.09–9.13	<0.001	
Model D								
Age	1.04	1.01–1.08	0.022	0.129	1.02	0.99–1.05	0.18	0.225
Tumor size	1.45	1.11–1.88	0.006		1.5	1.12–2	0.006	
Margin status	2.24	1.12–4.5	0.023		3.39	1.46–7.89	0.005	
LN status	2.39	1.07–5.35	0.034		5.51	2.71–11.2	<0.001	
Model E								
Age	1.04	1.01–1.08	0.017	0.133	1.02	0.99–1.05	0.236	0.202
Tumor size	1.45	1.12–1.88	0.005		1.43	1.09–1.89	0.011	
Margin status	2.21	1.09–4.46	0.028		3.31	1.44–7.61	0.005	
LNR (%)	1.02	1–1.04	0.017		1.07	1.03–1.11	0.001	

LN: lymph nodes; LNR (lymph node ratio) = (number of positive LN)/(total LN removed) ∗ 100.

**Table 3:** Cox regression multivariate analysis between pathologic features and overall survival.

Covariate	Number of patients	Hazard ratio	95% CI	*P*
Model A				
Age	206	1.02	1–1.04	0.019
Tumor size		1.31	1.16–1.48	<0.001
Margin status		2.12	1.5–3.01	<0.001
Model B				
Age	206	1.02	1–1.04	0.018
Tumor size		1.35	1.19–1.52	<0.001
Margin status		1.69	1.17–2.44	0.005
Lymphovascular invasion		1.96	1.37–2.82	<0.001
Model C				
Age	206	1.02	1–1.04	0.014
Tumor size		1.3	1.15–1.47	<0.001
Margin status		1.58	1.09–2.29	0.016
Perineural invasion		2.19	1.44–3.32	<0.001
Model D				
Age	206	1.02	1.01–1.04	0.008
Tumor size		1.27	1.12–1.43	<0.001
Margin status		1.75	1.23–2.5	0.002
LN status		2.44	1.64–3.64	<0.001
Model E				
Age	206	1.02	1–1.04	0.012
Tumor size		1.28	1.13–1.45	<0.001
Margin status		1.95	1.37–2.76	<0.001
LNR (%)		1.02	1.01–1.03	<0.001

LN: lymph nodes; LNR (lymph node ratio) = (number of positive LN)/(total LN removed) ∗ 100.

**Table 4:** Classification accuracy of CART analysis for 1-year and 3-year mortality.

	Training set				Testing set			
	Total cases	Dead	Alive	% Accuracy	Total cases	Dead	Alive	% Accuracy
1-year mortality								
Actual class								
Dead	56	29	27	52%	56	17	39	30%
Alive	151	10	141	93%	151	15	136	90%

		Overall % Accuracy		82%		Overall % Accuracy		74%

3-year mortality								
Actual class								
Dead	113	88	25	78%	113	85	28	75%
Alive	74	25	49	66%	74	25	49	66%

		Overall % Accuracy		73%		Overall % Accuracy		72%

CART: Classification and Regression Trees.
